# Nutritional self-management in chronic diseases: a conceptual analysis

**DOI:** 10.3389/fpubh.2025.1680903

**Published:** 2025-11-26

**Authors:** Lingzhu Zhang, Hongyan Li, Tingting Huang, Minhui Yang, Xinyan Yu, Yu Liu

**Affiliations:** School of Nursing, Jiangxi Medical College, Nanchang University, Nanchang, Jiangxi, China

**Keywords:** concept analysis, chronic diseases, healthcare, nutrition, self-management

## Abstract

**Aims:**

This study employs conceptual analysis to clarify the core definition of nutritional self-management in chronic diseases, addressing the critical issues of ambiguous conceptualization and the lack of operational standards in current nutrition management against the backdrop of increasing chronic disease burden. The findings aim to establish a scientific clinical evaluation framework and inform evidence-based intervention strategies.

**Methods:**

Walker and Avant’s concept analysis approach was used to analyze the concept of nutritional self-management in chronic diseases. A comprehensive literature search was conducted across PubMed, Web of Science, CINAHL and Embase databases, spanning from their inception to 2024.

**Results:**

A total of 52 articles were included after screening. Nutritional self-management in chronic diseases was defined by six attributes: (1) Dietary management adherence. (2) Establishing collaborative partnership between patients and healthcare providers. (3) Acquisition, evaluation and utilization of healthcare resources. (4) Decision-making and action. (5) Perception and adaptation to patient role. (6) Emotional regulation and management. The antecedent variables of nutritional self-management in chronic diseases were sociodemographic factors, nutritional self-management ability, opportunities, and motivation. The consequences mainly affected the patients from three dimensions: the individual, family and society, forming a virtuous cycle of “patient benefit-family stress reduction-social efficiency improvement.”

**Conclusion:**

This study systematically develops a conceptual model of nutritional self-management for chronic disease patients, grounded in classical Walker and Avant’s concept analysis. Through integrative analysis of common characteristics in chronic disease nutrition management, we propose a theoretically-grounded structured framework that establishes an essential foundation for developing standardized assessment tools and conducting clinical empirical research. While the core elements demonstrate cross-cultural applicability, their real-world implementation requires careful consideration of contextual moderating factors across different regions. We recommend future multi-center collaborative studies incorporating disease-specific characteristics to further validate the generalizability of this framework.

## 1 Introduction

Chronic diseases, also known as chronic non communicable diseases (NCDs), are caused by the combined effects of genetic, physiological, environmental and behavioral factors (1). The Centers for Disease Control (CDC) and Prevention in the United States defines chronic diseases as conditions that last at least 1 year, which requires ongoing medical attention or limit activities of daily living or both (2). The incidence of chronic diseases has been increasing rapidly with the continuous population growth and aging. Given their prolonged duration and gradual progression, chronic diseases have emerged as a major public health challenge worldwide (3, 4). According to the World Health Statistics Report (2023), chronic diseases caused approximately 40 million deaths globally in 2019, accounting for 74% of total deaths (5). Among these, cardiovascular diseases, cancer, chronic respiratory diseases, and diabetes mellitus (DM) emerge as the leading contributors to the global burden of disease, posing significant public health challenges. Chronic diseases compromise patients’ quality of life and impose significant economic and psychological burdens on families and societies (6, 7). The CDC reports that chronic diseases account for nearly 75% of total healthcare expenditures in the U.S., costing approximately $5,300 per person annually (8). Similarly, chronic diseases contributed to 91.0% of deaths and 86.7% of disabilities in 2021 in China (9).

With the integration of artificial intelligence and medicine, innovative treatment methods and novel drugs are emerging like mushrooms after rain. The average lifespan of chronic disease patients has been significantly prolonged through multidisciplinary and multi-team collaborative interventions including treatment, nursing and rehabilitation. Even patients with severe malignant tumors can achieve long-term survival (10–13). In the long-term disease management, patients need to collaborate with their families or healthcare providers to achieve effective behavioral self-management (nutrition, exercise and lifestyle, etc.), cognitive self-management (disease-related information/resources and psychological management) and medical self-management (treatment and complication management, etc.) (14, 15). Obviously, the focus of chronic disease management is shifting from hospital-centered care to community- and home-based care. To adapt to this shift, patients should gradually cultivate an active health awareness, develop strong health beliefs, and improve their self-management capabilities gradually to reduce the burden of their families and healthcare system (16, 17).

Self-management refers to the process by which patients actively engage in treatment and carry out daily health-related behaviors to manage chronic diseases (18, 19). It is the core component of chronic disease management (20). Through effective self-management, patients with chronic diseases can improve their disease awareness, slow disease progression, and optimize physical condition and functional capacity, thereby maintaining a positive mindset and enhancing their quality of life (21).

Nutritional self-management, as one of the intervention targets in chronic disease management, plays an important role in patients’ self-management. For chronic disease patients, nutritional self-management represents the most economical, effective, and applicable way to prevent complications and improve quality of life (22–24). Nutritional self-management is endowed with diverse conceptual connotations across disciplines, owing to variable research focuses and perspectives. Although the definition of nutritional self-management has not been clearly unified, the conceptual perspectives from different disciplines provide rich, multifaceted insights. In nutriology, nutritional self-management refers to the proactive process by which individuals assess, select, and adapt their dietary intake according to their specific nutritional requirements and health conditions, with the goal of maintaining or enhancing their overall nutritional status (25). In public health science, this concept is closely tied to community-level nutrition education and intervention programs, aiming to enhance public nutrition literacy and self-management skills, thereby fostering population health improvement (26). In clinical medicine and nursing, nutritional self-management is integral to disease management and functional rehabilitation. Patients collaborate with healthcare providers to implement home-based nutrition strategies, optimize disease control, and support recovery (27). It can be seen that different disciplines approach the concept of nutritional self-management from different perspectives, leading to a vague connotation of the concept. The ambiguity of this concept may lead to cognitive limitations and biases, hindering in-depth theoretical exploration and the advancement of evidence-based practices in chronic disease management (28–30).

This study employs the Walker and Avant’s concept analysis method to delineate the attributes of nutritional self-management in chronic diseases. The aim of this study is to develop a rigorously structured conceptual framework, characterized by well-defined parameters, to offer a standardized foundation for biomedical and interdisciplinary researchers examining nutritional self-management in this patient cohort.

## 2 Methods

### 2.1 Analytical methods

A concept analysis allows researchers to systematically investigate and articulate key characteristics of an abstract and hard-to-define phenomenon by identifying the key components and understanding how they might relate to other concepts. There are several methodologies available to execute a concept analysis. The Walker and Avant classical concept analysis methods are widely used in the literature due to their ease of understanding and executability. This methodology consists of 8 steps: selecting a concept, determining the aims of the analysis, selecting the literature, determining the attributes, identifying a model case, identifying additional cases, identifying the antecedents and consequences, and defining the empirical referents (31).

### 2.2 Search methods

#### 2.2.1 Data source

A comprehensive literature search was performed in PubMed, Web of Science, CINAHL, and Embase databases. The search terms included keywords and MeSH/Emtree terms related to three core concepts: (1) “Self-Management” OR “Self Care” OR “Self Management” OR “Management, Self,” (2) “Chronic Disease” OR “Chronic Diseases” OR “Chronic Illness” OR “Chronic Conditions,” and (3) “Nutrition” OR “Nutritional Self-Management.” These terms were combined using Boolean operators (AND between concepts, OR within synonyms), with field-specific syntax adapted for each database. An example of the core term combination is: (“Self-Management” OR “Self Care”) AND (“Chronic Disease” OR “Chronic Illness”) AND (“Nutrition”). The complete search strategies, including all query details and syntax adjustments, are provided in Supplementary Table S1. The search period covered records from the inception of each database to *July 31, 2025*. Other records were identified by manually searching the reference lists of the relevant studies. The inclusion criteria for the literature are as follows: (1) The subjects or participants were patients with chronic diseases, and the main research content was nutritional self-management. (2) The research is related to the attributes, evolution, antecedents, consequences and relevance to the topic of the concept. (3) Original research or conceptual papers published in English. Remove literature irrelevant to the topic, duplicate publications, or full texts unavailable, as well as systematic review studies to avoid evidence overlap.

#### 2.2.2 Data analysis

We used the Endnote 21.3 software to manage the references and remove duplicates. After the deduplication process, two reviewers (L.Z & H.L) screened the titles and abstracts of each study independently according to the inclusion and exclusion criteria. Any discrepancies were arbitrated by the corresponding author (Y.L) to achieve consensus. The same methodology was used to screen the full-texts of the studies.

#### 2.2.3 Search results

**Figure 1:**
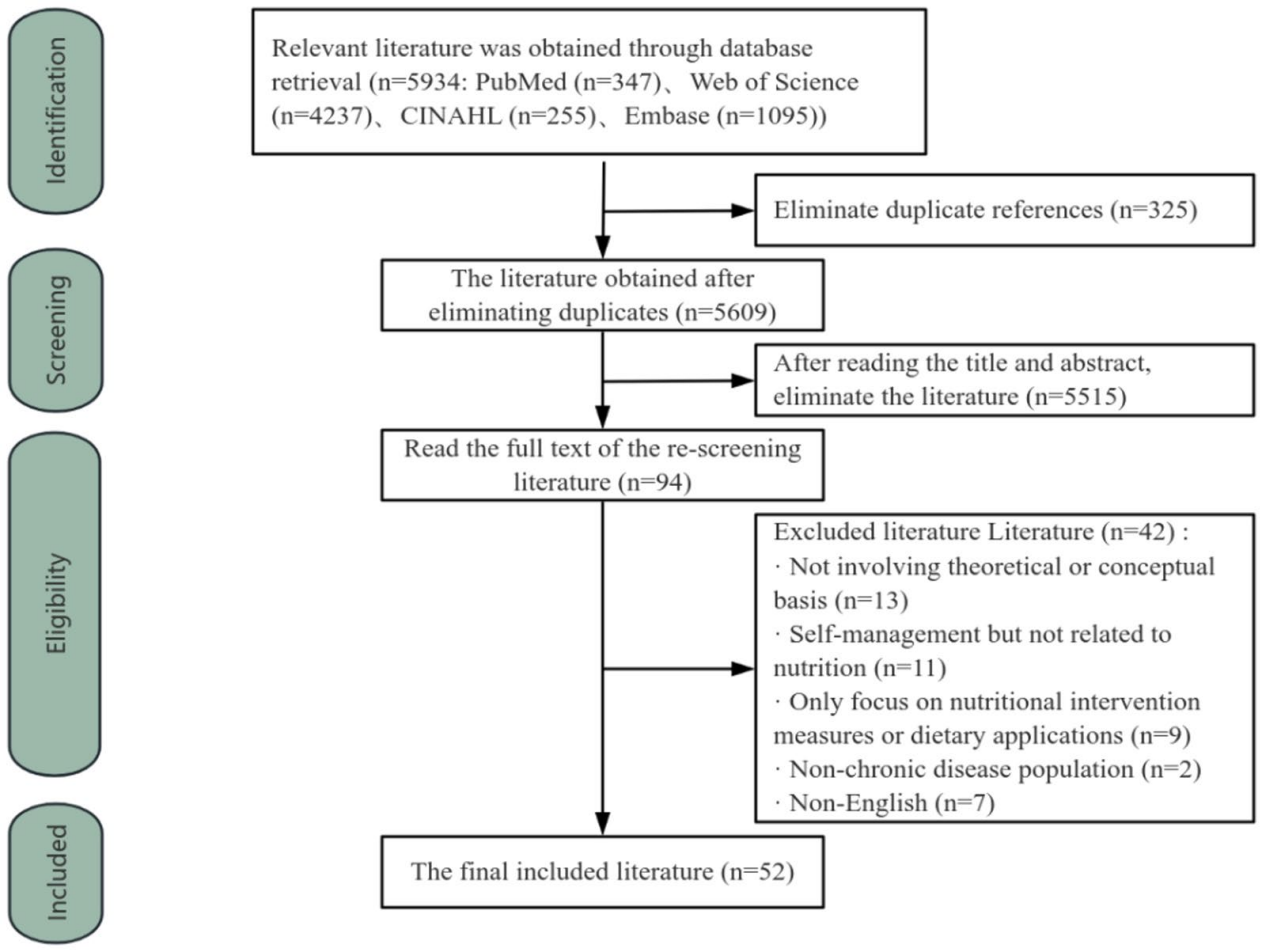
Flowchart of the article search and exclusion process of the concept analysis.

A total of *5,934* records were retrieved through database search. After removing duplicate records, *5,609* articles remained. By reading the titles and abstracts, *5,515* articles were excluded, and the remaining *94* articles had been fully read. Ultimately, 52 articles were selected for the concept analysis. The specific screening flowchart is shown in Figure 1. Then, the two reviewers (L.Z & H.L) extracted the data of each study including author, publish year, country, study design, target population, age range, sex, sample size as well as definition, attributes, antecedents and consequences of nutritional self-management in chronic diseases (see Tables 1, 2). The 52 articles were synthesized using a thematic synthesis approach (32). This method systematically codes data to create descriptive and analytical themes. It follows 3 steps: line-by-line coding, forming descriptive themes, and developing broader analytical themes that identify key insights beyond the original studies (see Table 3).

**Table 1:** Characteristics of the included studies.

Author (Year)/Country	Study design	Participants	Sample size	Age range (years)	Sex
Buxton (1985)/USA (40)	RCT	Patients with overweight	11	–	Male: 4 (36.4%) Female: 7 (63.6%)
Gillis (1995)/USA (61)	RCT	Renal insufficiency	840	–	–
Neumark-Sztainer (1998)/USA (99)	Cross-sectional study	Adolescents without CHRONIC ILLNESs. Adolescents with chronic illness.	9,343	–	No chronic illness (*n* = 8,322): Male: 4045 (48.6%) Female: 4277 (51.4%) Chronic illness (*n* = 1,021): Male: 438 (42.9%) Female: 583 (57.1%)
Quandt (1998)/USA (41)	Case study	Widows in the community	2	83	Female
McDonald (2000)/USA (43)	Case study	Widowers in the community	12	70–96	Male
Quandt (2000)/USA (42)	Qualitative research	Widows in rural areas	145	70–96	Female
Campbell (2008)/Australia (53)	RCT	Chronic kidney disease	47	–	Male: 29 (61.7%) Female: 28 (38.3%)
Gazmararian (2009)/USA (52)	Qualitative research	Diabetes	35	48–54	–
Agatha (2010)/South Africa (96)	RCT	Type 2 diabetes	51	40–65	Male: 21 (41.2%) Female: 30 (58.8%)
Savoca (2011)/USA (44)	Qualitative research	Older adults	635	≥60	Male: 291 (45.9%) Female: 344 (54.1%)
Dobbins (2012)/USA (77)	RCT	Diabetes	43	–	–
Vieira (2012)/Brazil (104)	Qualitative research	Chronic metabolic syndrome	9	37–63	–
Alameddine (2013)/USA (57)	Cross-sectional study	Type 2 diabetes	333	–	Male: 184 (55.3%) Female: 149 (44.7%)
Karavetian (2013)/USA (47)	RCT	Hemodialysis	122	–	Male: 60 (49.0%) Female: 62 (51.0%)
Ovayolu (2013)/Turkish (93)	Cross-sectional study	Cancer	260	≥18	Male: 117 (45.0%) Female:143 (55.0%)
Steers (2014)/USA (108)	Case study	Type 2 diabetes	1	61	Female
Elliott (2015)/USA (48)	Cross-sectional study	Hemodialysis	95	≥18	Male: 57 (60.0%) Female: 38 (40.0%)
Fort (2015)/Mexico (95)	Qualitative research	Hypertension and/or type 2 diabetes	9	–	Male
Kim (2015)/Korea (84)	Cross-sectional study	Chronic obstructive lung disease	751	>18	Male: 556 (74.0%) Female: 195 (26.0%)
Pan (2015)/China (75)	Cross-sectional study	Type 2 diabetes	244	41–84	Male: 110 (45.3%) Female: 134 (54.7%)
Ahn (2016)/Korea (112)	Interventional study	Diabetes	26	–	Male: 13 (50.0%) Female: 13 (50.0%)
Chen (2017)/China (46)	Quasi-experimental design	Older adults	120	≥65	Male: 36 (30.0%) Female: 84 (70.0%)
Wang (2017)/China (92)	Cross-sectional study	Chronic obstructive pulmonary disease	100	50–89	Male: 71 (71.0%) Female: 29 (29.0%)
Abel (2018)/New Zealand (50)	Qualitative research	Diabetes	20	43–69	Male: 10 (50.0%) Female: 10 (50.0%)
Baruth (2018)/USA (113)	RCT	Arthritis	321	–	Male: 39 (12.0%) Female: 282 (88.0%)
Hakami (2018)/Canadian (51)	Qualitative research	Chronic respiratory disease	93	–	Male: 49 (53.0%) Female: 44 (47.0%)
Kelly (2019)/Australia (63)	RCT	Chronic kidney disease	80	–	Male: 51 (64.0%) Female: 29 (36.0%)
Ko (2019)/USA (101)	Cross-sectional study	Diabetes	66	18–61	Male: 48 (72.7%) Female: 18 (27.3%)
Lim (2019)/Korea (78)	Cross-sectional study	Hemodialysis	145	–	Male: 68 (46.9%) Female: 77 (53.1%)
Lim (2019)/Singapore (98)	Cross-sectional study/qualitative research	Diabetes	433	–	Male: 221 (51.0%) Female: 212 (49.0%)
Warner (2019)/Australia (4)	Qualitative research	Chronic kidney disease	21	28–78	Male: 14 (67.0%) Female: 7 (33.0%)
Wong (2019)/USA (29)	RCT	Non-insulin-dependent diabetes mellitus	35	35–75	Male: 18 (51.4%) Female: 17 (48.6%)
Aga (2020)/USA (79)	Cross-sectional study/RCT	Type 2 diabetes and comorbid heart failure	180	–	Male: 118 (66.0%) Female: 62 (34.0%)
Al-Hadhrami (2020)/USA (94)	Cross-sectional study	Type 1 diabetes	210	–	Male: 62 (29.5%) Female: 148 (70.5%)
Haymer (2020)/USA (85)	Cross-sectional study	Prostate cancer	236	–	–
Kelly (2020)/Australia (62)	RCT	Chronic kidney disease	80	–	Male: 51 (64.0%) Female: 29 (36.0%)
Lim (2020)/Malaysia (97)	Qualitative research	Dialysis	253	–	–
Meadows (2020)/USA (71)	Qualitative research	Type 1 diabetes	12	15–17	Male: 5 (41.7%) Female: 7 (58.3%)
Guo (2021)/China (89)	Cross-sectional study	Chronic disease	19,291	≥45	Male: 9344 (48.4%) Female:9947 (51.6%)
Konerding (2021)/Germany (66)	RCT	Type 2 diabetes and/ or coronary heart disease	–	–	–
Leak (2021)/USA (87)	Qualitative research	Prediabetes	48	28–76	Male: 2 (4.2%) Female: 46 (95.8%)
Yang (2021)/China (107)	Cross-sectional study	Type 2 diabetes	358	≥29	Male: 165 (46.1%) Female: 193 (53.9%)
Bross (2022)/USA (49)	Cross-sectional study	Diabetes	99	–	Male: 31 (31.1%) Female: 68 (68.7%)
Goh (2022)/Singapore (74)	RCT	Chronic disease	250	–	Male: 78 (31.2%) Female: 68 (68.8%)
Hwang (2022)/USA (103)	Qualitative research	Hemodialysis	14	40–80	Male: 5 (35.7%) Female: 9 (64.3%)
Jones (2022)/USA (70)	Qualitative research	Hypertension	19	62–91	Male: 2 (12.9%) Female: 17 (87.1%)
Misra (2022)/Iran (73)	Cross-sectional study	Type 2 diabetes	820	≥18	Male: 337 (41.1%) Female: 483 (58.9%)
Ansong (2024)/USA (65)	Qualitative research	Stroke	–	–	–
Celik (2024)/Türkiye (76)	Cross-sectional study	Type 2 diabetes	387	18–82	Female
Chaudhry (2024)/USA (86)	Interventional research	Hemodialysis	8	–	–
Peng (2024)/China (45)	Cross-sectional study	Hemodialysis	445	–	Male: 260 (58.4%) Female: 185 (41.6%)
Scholl (2024)/Germany (102)	Qualitative research	Type 2 diabetes and/ or coronary heart disease	35	–	Male: 21 (60.0%) Female: 14 (40.0%)

**Table 2:** Attributes, antecedents and consequences of nutritional self-management in chronic diseases.

Components	Contents	References
Attributes		
Dietary management adherence	Adhere to healthcare professionals’ recommendations in implementing dietary self-management. Implement dietary management throughout the dynamic course of the disease. Closely linked to disease recurrence and progression.	(4, 47–53)
Establishing collaborative partnership between patients and healthcare providers	Establish long-term communication to enhance self-efficacy in chronic disease management. Utilize telemedicine and big data technologies to provide precision nutrition guidance. Encourage active participation from patients and families for efficient information sharing.	(4, 47–51, 57–63, 65, 66)
Acquisition, evaluation and utilization of healthcare resources	Proactively identify nutrition information tailored to individual needs through digital and physical channels. Filter credible content to avoid misleading pseudoscience and vague guidance. Translate resources into practical dietary strategies based on disease stage and personal conditions.	(4, 49–51, 70)
Decision-making and action	Patients face information conflicts and cognitive biases in self-adjusting diets. Structured decision support resolves decision-making dilemmas. Breaking goals into steps transforms complex management into actionable tasks. Positive decision-making experiences empower patients and sustain long-term adherence.	(4, 48–51, 70, 71, 73, 74)
Perception and adaptation to patient role	Patients should recognize their self-management responsibilities and strengthen nutritional control awareness. Adjust diet and social roles to achieve long-term disease stability.	(49–51, 75–77)
Emotional regulation and management	Psychological regulation mitigates negative emotions from dietary restrictions, enhancing resilience and treatment adherence. Psychological support combined with medical management improves both mental and physical health outcomes.	(49, 78, 79)
Antecedents		
Sociodemographic factors	High-risk groups: Men, older individuals, those widowed/living alone, and rural residents have poorer self-management of nutrition and worse disease outcomes. Protective factors: Patients with higher education, better financial status, and stable jobs exhibit stronger self-management skills. Core issue: Accessibility to resources (medical support, health information) is critical, with rural areas facing higher risks of chronic disease complications due to limited resources.	(50, 70, 79, 84–87, 89)
Nutritional self-management ability	Nutritional literacy. Practical skills. Cognitive/psychological regulation abilities.	(48, 49, 51, 52, 65, 70, 73, 78, 87, 92–96)
Nutritional self-management opportunities	Physical opportunities. Social opportunities.	(4, 47, 50, 57, 63, 70, 71, 79, 96, 98, 99, 101, 102)
Nutritional self-management motivation	Dietary habits. Negative emotions. Self-efficacy.	(48–51, 72, 101, 103, 104, 107)
Consequences		
The individual level	Promote the development of healthy behaviors and improve physical fitness. Enhance self-management efficacy and improve psychological state.	(4, 51, 53, 61, 74, 76, 104, 108)
The family level	Home-based collaborative disease management. Providing daily care and dietary support.	(73, 98, 104, 108)
The social level	Save medical expenses. Cut waste and improve resource allocation.	(62, 63, 108, 112, 113)

**Table 3:** Thematic synthesis.

Coding step	Key operations	Application
Step one: line by line coding	Annotate meaningful units in the text. Preserve raw phrasing, generate initial codes.	Sections of text were assigned a label and, via the use of constant comparison code, codes were developed.
Step two: development of descriptive themes	Group similar codes. Identify patterns and relationships among codes.	Data which appeared similar was grouped, thematic networks were developed to assist in theme examination.
Step three: development of analytical themes	Development of new insights based on themes.	The structure of each theme was reviewed and reduced if similar characteristics were apparent.

## 3 Results

### 3.1 Confirmation of concepts

In accord with Walker and Avant’s method, we identified as many uses of the concept as possible. “[If] the analysis is done well, the defining characteristics should immediately call the concept to mind.” To achieve this, dictionaries, thesauruses, and available literature may be utilized (31).

#### 3.1.1 The literal interpretation of the concept

There is no direct definition of nutritional self-management in Merriam-Webster’s Advanced Learner’s English Dictionary, where “Nutrition” is defined as “the act or process of nourishing or being nourished. Specifically: the sum of the processes by which an animal or plant takes in and utilizes food substances.” “Self-management means finding the self-control and mastery needed to control one’s work.” The definition of “Self-management” in the MeSH glossary is “Individual’s ability to manage the symptoms, treatment, physical and psychosocial consequences and lifestyle changes inherent in living with a chronic condition. Efficacious self-management encompasses ability to monitor one’s condition and to affect the cognitive, behavioral and emotional responses necessary to maintain a satisfactory quality of life.”

#### 3.1.2 The application of concepts in literature

The concept of self-management was first proposed by Albert Bandura in the 1960s based on social learning theory and is closely related to the concept of self-control (33). In the field of healthcare, the origin of the self-management concept can be traced back to a study in 1971 that aimed to verify the effectiveness of smoking cessation training (34). Building upon Bandura’s theoretical framework, Manz et al. argued that the process of self-management was driven by the realization of anticipated goals and is influenced by both internal (personal controllable) and external (personal uncontrollable) factors (35). Building upon theoretical advances, the self-management model proposed by Lorig and Holman in 2003 emerged as a seminal contribution to chronic disease self-management research. Their influential framework established three fundamental dimensions of patient self-management: medical management, role management, and emotional management, while simultaneously identifying five core competencies essential for effective execution: problem solving, decision making, resource utilization, the formation of a patient-provider partnership and action planning (25). Subsequently, researchers gradually discovered the positive benefits of self-management in improving individuals’ cognition and behaviors, enhancing quality of life, alleviating clinical symptoms and optimizing the use of medical resources (36). Currently, self-management is recognized as a promising strategy to mitigate the mounting pressure on health and social-service systems, stemming from workforce shortages, rising healthcare demands, population growth, and budgetary limitations.

As self-management practices continue to evolve, their conceptual framework has expanded substantially, branching into specialized domains such as nutritional management, functional rehabilitation, medication adherence, psychological and emotional regulation, self-monitoring of health status, and symptom recognition and coping (11, 37). The Global Burden of Disease Study has shown that the core indicator of Disability-Adjusted Life Years (DALY) is mainly attributed to behavioral risk factors such as malnutrition, reduced intake of fruits and vegetables, increased Body Mass Index (BMI), smoking, excessive alcohol consumption, and low physical activity levels (38, 39). Evidently, these behavioral risk factors are predominantly associated with nutritional self-management, underscoring the critical role of dietary behavior regulation in disease prevention and health promotion.

The concept of nutritional self-management was first introduced in 1985 as part of a health management program for weight loss behavioral therapy in overweight patients. Studies revealed that relying solely on caloric restriction failed to help overweight patients achieve a balanced diet. However, after implementing a nutritional self-management program, patients demonstrated reduced total caloric intake, weight loss, and improved nutritional status (40). Since then, nutritional self-management has received increasing attention. In 1998, Quandt et al. proposed that the nutritional status of older populations in communities was determined by a series of nutrition-related behaviors (41). These behaviors facilitate the effective acquisition, utilization, and safe maintenance of food resources, collectively constituting essential nutritional self-management strategies. Given this, Quandt proposed a conceptual model of nutritional self-management. This model prompts that individuals promote their nutritional plans by synthetically utilizing self-care, informal support, formal support and healthcare resources. Guided by the theoretical framework, in-depth interviews were conducted with 64 rural older widows to examine determinants of food acquisition, utilization, and safety, while simultaneously exploring their engagement with self-care practices and support systems-including informal and formal supports and healthcare resources. Women with high nutritional risk were accurately captured, providing clear direction for nutritional behavior interventions at the community and family levels (42). The nutritional self-management conceptual model has since been adopted in subsequent studies (43, 44). Robust evidence reveals significant gaps in nutritional self-management support for chronic disease patients. Current mainstream interventions (e.g., clinical guidelines and online resources) exhibit two fundamental limitations: (1) the absence of a systematic framework reduces chronic disease nutrition management to rigid dietary guidelines, failing to address its fundamental requirement for ongoing monitoring and adaptive adjustments, and (2) over-reliance on unidirectional information delivery without incorporating critical empowerment elements (knowledge, skills, and self-confidence). This “information-over-management” bias reflects conceptual immaturity in the field. Although existing research has investigated nutrition management theories across various chronic conditions, consensus remains lacking regarding standardized operational definitions and implementation pathways (29, 30, 45, 46).

### 3.2 Definition of concept

Defining attributes are the most relevant characteristics associated with a concept. Through literature review and analysis, six conceptual attributes are identified to define the nutritional self-management in chronic diseases (see Table 2).

#### 3.2.1 Dietary management adherence

Dietary management adherence is an important defining attribute of nutritional self-management in chronic diseases and a major focus in this research field. In these patients, adherence refers to the level of dietary self-management achieved in accordance with healthcare professionals’ recommendations (4, 47, 48). Patients must integrate dietary management throughout the process of food selection, preparation, cooking and consumption, in line with the dynamic characteristics of their diseases (49, 50). Extensive research demonstrates that high adherence to dietary management significantly reduces complications, enhances self-care capacity, and improves quality of life (29, 51).

Dietary management adherence for patients with chronic diseases is closely related to disease recurrence and progression, holding an extremely important position in disease management (52, 53). It is confirmed that long-term adherence to the Mediterranean diet provides multiple health benefits in cardiovascular disease patients. This dietary pattern aids in lowering lipids, reducing inflammation, inhibiting platelet aggregation, and mitigating oxidative stress, thereby reducing the recurrence of cardiovascular events and improving quality of life (54). Similarly, prospective studies in gestational diabetes mellitus (GDM) prompt better adherence enhanced glucose tolerance and is associated with a lower incidence of GDM (55).

#### 3.2.2 Establishing collaborative partnership between patients and healthcare providers

Establishing patient-provider partnership enhances self-management capabilities and self-efficacy in chronic disease patients (47, 49, 56, 57). Healthcare providers should establish and maintain long-term communication with patients and families (48). They should encourage patients to participate in social activities, and maintain close interactions with their family and friends (50, 58), helping them more willing to express and share their own feeling in the daily self-management process (51, 59–61).

Moreover, healthcare providers could use remote medical service platforms to provide patients with dynamic personalized guidance (4, 62, 63) and nutritional counseling (47). The user profiling technology under big data algorithms, enables the provision of precise home services (64). Hence, disease-related nutritional self-management information and technical support become more accessible to the patients, strengthening their self-efficacy in nutritional self-management (65). Similarly, chronic disease patients and their families should also actively interact and cooperate with healthcare providers to ultimately achieve two-way communication (66).

#### 3.2.3 Acquisition, evaluation and utilization of healthcare resources

Resource acquisition, evaluation, and utilization encompass a chronic disease patient’s capacity to actively identify, critically assess, and effectively apply appropriate nutritional self-management resources tailored to their individual needs (4, 51, 67, 68). Nutrition-related information and disease-specific resources can be acquired through both digital (e.g., online platforms) and physical (e.g., hospitals, communities, residential settings, and patient associations) channels. Additionally, patients must critically assess the vast array of available information and resources to mitigate exposure to misinformation or ambiguous content (49, 50, 69, 70). With advancing disease literacy, patients with chronic diseases progressively develop the capacity to systematically curate and synthesize accessible resources, thereby optimizing their nutritional self-management strategies in alignment with disease-specific pathophysiology and personalized dietary requirements.

#### 3.2.4 Decision-making and action

Another important attribute of nutritional self-management in chronic diseases is the decision-making and action. Unlike the acquisition and utilization of resources, which focuses on external support systems, this attribute emphasizes the internal process through which patients translate cognitive assessments into concrete nutritional management behaviors. Nutritional self-management in chronic diseases is an ongoing journey, and patients often face various hurdles along the way. Under the premise of limited extended medical support, obtaining disease-related nutritional management guidance from professionals at any time is restricted. Patients must independently determine appropriate dietary and supplemental adjustments based on their individual decision-making capacity (50, 51, 71, 72).

However, in the long-term nutritional self-management, patients often fall into decision-making dilemmas, including information dilemma (insufficient or conflicting information due to divergent clinician-patient perspectives), trade-off dilemma (conflict of role attributes or correction of behavioral habits) and conceptual dilemma (delayed decision-making driven by prior experiences or cognitive biases such as the framing effect) (4, 73). Effective patient-provider communication is crucial for overcoming decision-making dilemmas in nutritional self-management (70). Healthcare professionals should use a patient-centered approach to guide patients in evaluating options (benefits, risks, drawbacks), provide structured decision support, and address confidence barriers. Positive decision-making experiences enhance patient autonomy, meanwhile breaking down complex self-management tasks into smaller, achievable steps can sustain motivation and foster gradual behavioral change (48, 49, 74).

#### 3.2.5 Perception and adaptation to patient role

Role perception and adaptation for chronic disease patients involves adopting and sustaining a healthy lifestyle to stabilize their conditions, while redefining their familial and societal roles, forming a critical component of nutritional self-management (49, 51, 75). Effective role perception during nutritional self-management requires patients to recognize its necessity, proactively assume self-management responsibilities, collaborate with healthcare professionals to implement personalized nutrition plans (50, 59), and concurrently adapt work commitments, preserve social connections with relatives, friends, and fellow patients, and integrate evolving social roles (76, 77).

#### 3.2.6 Emotional regulation and management

Emotional regulation and management of chronic disease patients involve the application of psychological coping strategies and behavior adjustments to effectively identify, accept and address negative emotions stemming from long-term dietary restrictions, ultimately improving psychological resilience, mental well-being, and disease self-management capabilities (49, 59).

Chronic disease patients often confront significant psychological distress due to prolonged dietary restrictions, unpredictable disease progression, and challenging treatment regimens, leading to anxiety, depression, and poor adherence behaviors. Without proper intervention, these emotional difficulties can worsen disease outcomes, particularly for patients with limited coping abilities or inadequate social support. Effective management requires integrating psychological approaches such as cognitive-behavioral strategies, mindfulness training, and structured support systems into standard clinical care. By addressing both emotional and medical needs, these interventions can enhance patients’ self-management capacity and improve long-term health results (60, 78, 79).

### 3.3 Cases

The purpose of the case construction is to provide clear real-life cases for the attributes, antecedents and consequences of the concept, and be able to express the application of this concept in the discipline (31). The following section provides the model, borderline and contrary cases.

#### 3.3.1 Model case

Ms. Zhang, 55 years old, is a retired worker with a junior high school education. She has suffered from hypertension and type 2 diabetes mellitus (T2DM) for 5 years. She occasionally experiences symptoms such as headaches, dizziness, and palpitations, which can be alleviated with rest. However, due to a lack of awareness regarding standardized management, she missed medications and neglected condition monitoring. Moreover, she mistakenly assumes that merely taking medication alone is enough to manage her condition. During a routine free check-up at her community health center, Ms. Zhang discovers that she has high blood pressure and elevated blood sugar. After consulting her doctor, she realizes that her inconsistent medication use and poor dietary habits have worsened her condition. The doctor cautions that failing to address these issues may lead to serious heart problems or stroke. Under the advice of professionals, Ms. Zhang begins to proactively learn about nutritional self-management and regularly communicate with medical staff about any confusion or problems. With their guidance, she formulates and implements a daily diet plan, and carries out nutritional self-management under the supervision of her family and friends. She also regularly monitors and records her blood pressure and blood sugar. In addition, Ms. Zhang actively participates in chronic diseases exchange meetings, sharing experiences and encouraging each other with fellow patients. She also learns how to identify and handle emergencies such as hypoglycemia and hypertensive crisis. After 2 months of consistent efforts, Ms. Zhang observes that her blood pressure and blood glucose levels are well controlled, accompanied by an improvement in her overall physical condition. She feels more energetic, and no longer needs to frequently visit the hospital or be hospitalized, nor dose she worry about a sudden deterioration of her condition.

#### 3.3.2 Borderline case

Mr. Wang, 70 years old, is a retired literature professor. He has suffered from DM and hypertension for nearly 10 years. After retirement, his social interactions have decreased, and Mr. Wang has gradually become taciturn and is not willing to communicate actively with medical staff. Mr. Wang believes he is knowledgeable. He often critically accepts the suggestions of medical staff and does not fully follow them. He also strongly believes in traditional Chinese medicine, frequently looking for ancient Chinese herbal prescriptions and refusing Western medication, even skipping prescribed doses. Despite recognizing the critical role of dietary management, Mr. Wang struggles to maintain a consistent diet plan owing to his solitary living situation. Recently, Mr. Wang learned that fasting and exercise could potentially extend lifespan. However, he adopted an extreme regimen-consuming only one meal per day for three consecutive days while discontinuing his prescribed medications, coupled with a strict daily 20,000-step walking routine. This ultimately resulted in hypoglycemia-induced syncope during a hike After emergency treatment, he recovered. But he felt that he had caused trouble for his family and medical staff and felt useless, often experiencing a sense of guilt.

#### 3.3.3 Contrary case

Mr. Li, a 52-year-old senior executive, was diagnosed with familial hypercholesterolemia at age 23 and developed hypertension 5 years ago. Despite having ample access to disease-related information, he lacks a clear understanding of nutritional self-management requirements and passively relies on dietitians’ plans without proactive engagement. Mr. Li can adhere to supervised health plans temporarily under a care manager’s guidance. However, his demanding work schedule and frequent business engagements have led to irregular eating habits and repeated consumption of high salt/sugar foods. He quickly reverts to previous habits, leading to inconsistent disease management. This cyclical pattern of relapse has disrupted both his professional performance and personal well-being, leaving him increasingly frustrated. Consequently, his blood pressure and blood glucose levels remain uncontrolled with signs of progressive deterioration. Ultimately, poor metabolic control leads to complications requiring hospitalization.

### 3.4 Antecedents

This study adopts the COM-B (Capability, Opportunity, Motivation-Behavior) model of behavior diagnosis as the theoretical framework for antecedent analysis (80). Originating from Michie et al.’s Behavior Change Wheel (BCW) theoretical system, this framework aims to systematically examine the multifaceted factors influencing nutritional self-management behaviors in chronic diseases patients. Widely applied in health behavior research (81), the model posits that individual behavior results from the interaction among three core components: capability, opportunity, and motivation. Building upon these core dimensions of the model and considering the unique characteristics of chronic disease nutritional management, we comprehensively analyzed factors affecting patients’ nutritional self-management while incorporating sociodemographic factors as important background variables. The subsequent sections will sequentially explore these factors, with all classifications remaining consistent with the theoretical structure of COM-B and supported by empirical evidence from relevant studies (82, 83). A summary of the influencing factors analyzed in this section is presented in Table 2.

#### 3.4.1 Sociodemographic factors

Sociodemographic factors include gender (56, 84, 85), age, marital status, occupation, educational level (86), family economic status (50, 87) and geographical location of residence (70). Male, older, widowed, or living alone patients usually face more significant challenges in nutritional self-management (79, 85, 88). Aged patients prone to have lower learning and cognitive abilities, as well as lower decision-making and action capabilities. Additionally, widowed and solitary patients are often introverted and may have trouble in keeping touch with healthcare and resources providers, resulting in low resource acquisition and utilization and poor compliance. Patients with stable jobs, higher education levels, and better economic status tend to have stronger proactive health awareness, as well as greater access to medical resources and chronic disease management support. Consequently, these patients typically demonstrate better self-management behaviors (89). Moreover, residential location significantly influences patients’ access to resources for nutritional self-management. This limitation is particularly pronounced in rural areas, where inadequate nutritional self-management contributes to delayed disease diagnosis, frequent disease recurrence, and poorer clinical outcomes. Consequently, rural populations exhibit higher disability and mortality rates associated with chronic diseases, along with generally unfavorable prognoses (70).

#### 3.4.2 Nutritional self-management ability

Ability refers to the knowledge, skills and psychological qualities required for an individual to perform specific behaviors. It includes both physical ability and mental ability (80). The nutritional self-management ability of the chronic disease patients encompasses nutritional literacy, practical skills and cognitive/psychological regulation abilities.

Nutritional literacy is defined as the ability to acquire, handle and understand nutritional information, as well as the skills required to make appropriate nutritional decisions (90). Chronic disease patients with high nutritional literacy actively seek nutritional information and adhere to self-management strategies. However, studies indicate that most chronic disease patients have weak nutritional literacy (49, 70). These patients often misunderstand disease-related dietary guidelines or struggle with conflicting information, impairing effective self-management (51, 65, 73, 91).

Nutritional practical skills refer to the ability to convert nutritional knowledge into specific skills or behaviors, including food selection, cooking techniques, and dietary plan adherence. These skills help slow disease progression, empower patients, and enhance quality of life. However, many patients with chronic diseases lack adequate nutritional practical skills due to disease-related or environmental limitations (48, 70, 78, 92–94). To effectively address this critical gap, a multidimensional support system incorporating structured family engagement, professional medical supervision, and tailored intervention strategies demonstrates significant potential for optimizing patients’ nutritional self-management outcomes (95, 96).

Cognitive and psychological regulation ability refers to the psychological resilience to cope with dietary temptations, emotional eating, and long-term behavioral changes. This core competency exhibits a significant “knowledge-action disconnect” phenomenon. Although patients generally recognize the importance of healthy eating, high-calorie food temptations, emotional fluctuations and impulsive decisions often triumph over reason, leading to frequent interruptions or termination of diet plans. A qualitative study points out T2DM patients choose to binge on high-sugar foods when encountering negative emotions, even though they are aware its harm (52). These findings suggest that effective nutritional self-management in chronic diseases demands not only cognitive-to-behavioral skill development but also enhanced psychological resilience, thereby holistically improving self-management capabilities.

#### 3.4.3 Nutritional self-management opportunities

Opportunity encompasses all extrinsic factors-ranging from material resources in the physical environment to socio-cultural support systems-that may enable or constrain behavioral outcomes (80).

Physical opportunities refer to instrumental services provided by publicly funded programs and institutions as well as private community organizations, which includes material circumstances such as time and resources that facilitate the behavior. For example, the information resources and venues provided by community health institutions, as well as the personalized nutritional management realized by the intelligent remote disease monitoring platform (63, 67). However, at present, there are challenges such as information overload, imperfect intervention plans of intelligent platforms (68, 97, 98), and insufficient policy and economic support, which directly affect patients’ nutritional self-management (57, 59, 99). Besides the above professional support, physical opportunities also include community supporting living service institutions, such as community stores providing convenient shopping and door-to-door delivery services, and restaurants or canteens providing customized meals for diseases (70).

Social opportunities refer to opportunities provided by interpersonal influence, social culture, and ways of thinking, including social and cultural norms, social support and other factors. Patients with chronic diseases can obtain nutritional self-management social support from healthcare professionals (68, 72, 100), family members and friends (50, 56, 79, 98, 99), and fellow patients (67, 69, 71, 101, 102), which directly affects patients’ nutritional self-management behavior. Research indicates that social support fosters positive nutritional management behaviors-family participation enhances adherence, while peer support drives behavioral improvement (69, 71). Trust-based interactions with healthcare professionals enable patients to receive tailored guidance and emotional support, facilitating personalized nutrition plans (4, 47, 96). However, there are currently problems such as the lack of a long-term communication mechanism and insufficient nutritionist resources, which restrict the effect of patients’ nutritional self-management (56).

#### 3.4.4 Nutritional self-management motivation

Motivation refers to all the brain processes that stimulate and guide behaviors. It encompasses habitual processes, emotional responses, and analytical decision-making (80). Highly motivated chronic disease patients exhibit greater initiative in learning nutritional strategies, managing dietary intake, and adjusting behaviors based on disease status.

Dietary habits, as a potential medium of autonomy, can become a source of vulnerability in the process of coping with chronic diseases (50, 51, 103). During the long-term treatment of chronic diseases, some patients are influenced by their family, culture and beliefs, leading to significant behavioral inertia and cultural appropriateness conflicts in the reconstruction of dietary behaviors, which in turn become obstacles to nutritional self-management (29, 56, 104).

Negative emotions is harmful for the willingness of nutritional self-management. Chronic disease patients are prone to negative emotions caused by decline in physiological functions, uncertainty about the prognosis of the disease and concerns about becoming a burden to their families. Some patients may experience frustration, depression, fear, and social isolation, resulting in a passive attitude toward disease treatment, significantly hindering their adherence to nutritional self-management (49, 50, 72).

Meanwhile, negative emotions directly reduce patients’ self-efficacy through cognitive resource depletion and coping efficacy frustration. Self-efficacy is a cognitive concept that refers to an individual’s confidence in controlling their behavior to achieve goals (105). Nutritional self-management efficacy reflects an individual’s tendency and motivation to activate, execute, and persist in executing nutritional self-management in difficult situations (106). Empirical evidence consistently indicates that self-efficacy significantly predicts nutritional self-management behaviors in chronic diseases (48, 101, 107). Specifically, diabetic patients with higher self-efficacy demonstrate stronger motivation and adherence to prescribed nutritional plans (101). As a core mediator, self-efficacy is a key predictor of behavioral change, and interventions need to simultaneously optimize social and cultural adaptability and enhance psychological resilience to achieve long-term improvement in nutritional management.

### 3.5 Consequences

#### 3.5.1 The individual level

##### 3.5.1.1 Promote the development of healthy behaviors and improve physical fitness

There is a saying in China that “a long illness makes a doctor.” Through proactive acquisition of evidence-based nutritional knowledge, multidisciplinary collaboration, and self-monitored plan adjustments, chronic disease patients establish clinically beneficial nutritional self-management practices. This will help them integrate the process of nutritional self-management into their daily lives, forming and gradually adapting to a new lifestyle (51, 53, 59, 74, 104, 108). Moreover, nutritional self-management is conducive to the auxiliary treatment and functional rehabilitation of chronic diseases, and plays a significant role in comprehensively enhancing the overall physical fitness of patients. A balanced diet enhances immune function, improves disease resistance, and optimizes metabolic regulation, thereby reducing acute exacerbations of chronic conditions triggered by factors such as infections. Sustaining appropriate dietary habits not only mitigates the risk of disease deterioration but may also decelerate the natural progression of certain chronic illnesses (61, 109). It also helps improve self-care abilities, facilitate patients’ reintegration into society and enhance their quality of life (4, 110).

##### 3.5.1.2 Enhance self-management efficacy and improve psychological state

Evidence-based nutritional self-management empowers chronic disease patients to cultivate higher level of health literacy, enhance disease perception, and sustain psychological resilience through positive behavioral adaptation. Psychological resilience empowers patients to maintain hopeful engagement in self-management, enhances their ability to navigate challenges, and mitigates stress-related distress (110). In addition, nutritional self-management helps maintain the nervous and immune system stable, which is the keyguard of homeostasis of long-term survival for chronic disease patients (53, 76, 108, 109).

#### 3.5.2 The family level

Families serve as key functional units in nutritional self-management, effectively controlling chronic disease progression, lowering hospital admissions and healthcare expenditures, and lessening the family’s overall burden (73). Family members play a crucial role in the process of nutritional self-management and are the main source of social support for patients. By fully participating in the process of patients’ nutritional self-management, family members could get a deeper understanding of the patients’ conditions and nutritional needs. They will provide companionship, daily life support and emotional support to the patients and improve the overall dietary habits and lifestyles of the family. This can effectively reduces the multifaceted negative impacts of chronic conditions through enhanced family cohesion and harmonious functioning (72, 98, 104).

#### 3.5.3 The social level

In 2019, NCDs affected 4.1 billion people (52% of global population) (111). Given this substantial burden, nutritional self-management proves to be a cost-effective strategy by improving disease control and reducing medication dependence, complications, and hospitalization rates, thereby lowering healthcare costs (68, 108, 112, 113). Recent U.S. models estimate that from 2020 to 2025, routine albumin-to-creatinine ratio testing combined with targeted nutritional interventions could prevent chronic kidney disease progression in about 1.3 million patients, reducing medical costs by roughly $16 billion (114). Moreover, nutritional self-management enhances healthcare efficiency by reducing unnecessary resource expenditure, allowing for optimal allocation of medical resources. This reallocation enables greater investment in high-impact areas such as pharmaceutical innovation, emergency/critical care, and preventive medicine, thereby improving overall healthcare cost-effectiveness (62, 63).

It must be particularly emphasized that achieving these benefits requires establishing localized implementation frameworks that accommodate regional variations in healthcare infrastructure and socioeconomic factors. Although nutritional self-management has proven its complementary value within integrated chronic disease prevention and control systems, its comprehensive economic impact still warrants further validation through long-term cost-effectiveness analyzes across different healthcare systems. Collectively, nutritional self-management demonstrates clinically significant benefits across individual, familial, and societal dimensions, with key outcomes systematically synthesized in Table 2.

### 3.6 Empirical referents

Empirical assessment is conducive to further clarification of concepts and measurement, providing more explicit and intuitive indicators for nursing practice. Nutritional self-management in chronic diseases is a multi-faceted concept. Given the heterogeneity of chronic conditions, existing assessment indicators primarily evaluate three key dimensions: dietary adherence, nutritional literacy and disease-specific self-efficacy. No currently available tool adequately assesses all critical dimensions of nutritional self-management in chronic diseases. The following are the existing measurement tools and the corresponding measurement attributes of the concept (115–118), as shown in Table 4.

**Table 4:** Empirical assessment indicators for nutritional self-management in chronic diseases.

Assessment tool	Attribute 1	Attribute 2	Attribute 3	Attribute 4	Attribute 5	Attribute 6
Perceived Dietary Adherence Questionnaire (PDAQ) (115)	FA	–	–	FA	PA	–
Renal Adherence Behavior Questionnaire (RABQ) (116)	FA	PA	–	FA	PA	PA
Nutrition Literacy Assessment Instrument (NLAI) (117)	PA	–	FA	FA	–	–
Nutrition Self-efficacy Questionnaire (NSEQ) (118)	FA	PA	FA	FA	PA	–

Attribute 1, Dietary management adherence; Attribute 2, Establishing collaborative partnership between patients and healthcare providers; Attribute 3, Acquisition, evaluation and utilization of healthcare resources; Attribute 4, Decision-making and action; Attribute 5, Perception and adaptation to patient role; Attribute 6, Emotional regulation and management; FA, fully assessed; PA, partially assessed; −, unassessed.

The absence of standardized instruments capable of comprehensively evaluating all critical dimensions of nutritional self-management in chronic diseases stems from 3 principal limitations. First, conceptual frameworks are often discipline-specific, leading to a narrow focus: nutritional tools prioritize objective biomarkers (e.g., dietary records), while psychological measures overemphasize self-efficacy without addressing cross-dimensional interactions (118). Second, traditional methods fail to track dynamic behaviors effectively. For example, standard questionnaires miss dietary changes during acute COPD flare-ups (119). Third, clinical convenience often favors easily administered tools (e.g., food frequency questionnaires), neglecting scientifically important factors like emotional regulation (120).

Future development priorities should focus on three key areas: integration of cross-disciplinary theoretical frameworks to construct dynamic assessment models, innovative applications of emerging technologies incorporating digital phenotyping and biosensor methodologies, and optimization of cross-cultural adaptability (86). Implementation strategies should emphasize short-term modular assessment system development that effectively combines core evaluation dimensions with disease-specific indicators, while long-term objectives require the integration of wearable medical devices and artificial intelligence technologies to establish personalized risk prediction models (121). Special consideration must be given to avoiding technocentric biases, necessitating alternative assessment approaches for special populations such as cognitively impaired individuals and the development of a closed-loop management system spanning evaluation, intervention, and re-evaluation processes (102).

## 4 Discussion

Global health is facing mounting pressure as chronic NCDs surge in prevalence, disability, and mortality rates. Evidence suggests that the prevailing disease management strategy, emphasizing pharmaceutical treatment and sporadic medical oversight, fails to adequately mitigate disease worsening or disability accumulation. While these protocols emphasize pharmaceutical symptom suppression, they systematically neglect evidence-based nutritional modifications that directly address pathophysiological drivers (122, 123). Global disease burden data shows that dietary risks cause 11 million deaths, accounting for 60% of NCDs-related deaths (111). A fundamental reconceptualization of chronic disease management is underway, placing nutritional self-management at its core to achieve breakthrough improvements in prevention and control (124, 125). However, the absence of a standardized, evidence-based framework for nutritional self-management hinders both research translation and clinical implementation, underscoring an urgent need for conceptual clarity in developing actionable solutions.

**Figure 2:**
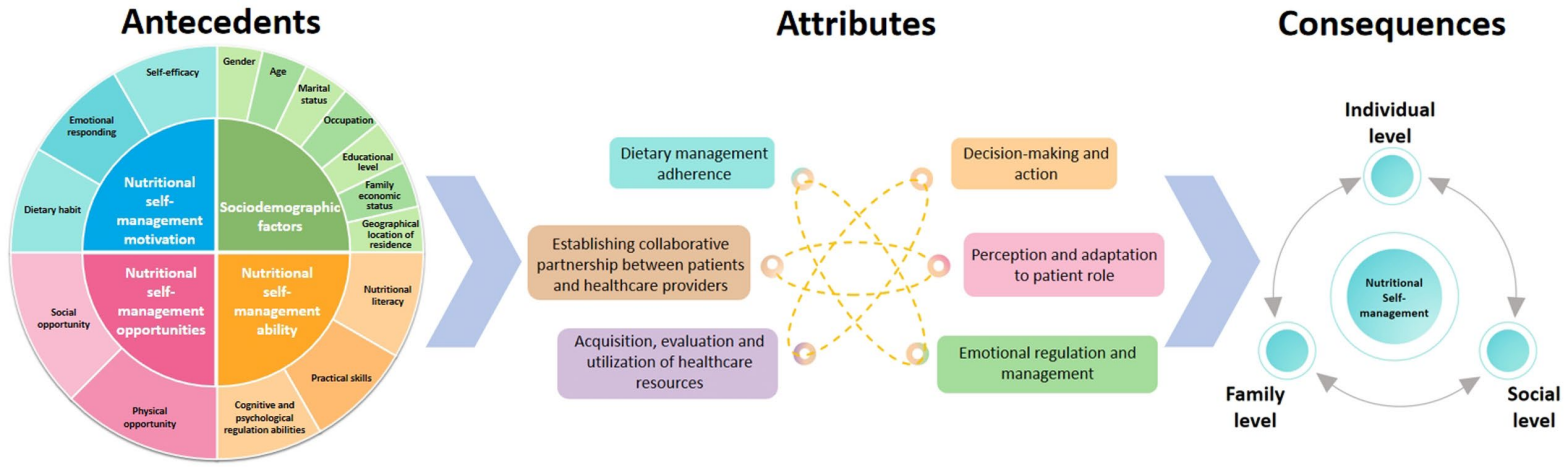
Conceptual diagram of nutritional self-management in chronic diseases.

This study proposes an operational definition and conceptual framework for nutritional self-management in chronic diseases to address current ambiguities in clinical practice. We identify six core attributes: (1) Dietary management adherence. (2) Establishing collaborative partnership between patients and healthcare providers. (3) Acquisition, evaluation and utilization of healthcare resources. (4) Decision-making and action. (5) Perception and adaptation to patient role. (6) Emotional regulation and management. The interrelationships among the antecedents, attributes and consequences of the nutritional self-management is illustrated in Figure 2.

This conceptual framework is rooted in the synergistic integration of self-management theory (25) and nutritional self-management models (41). Through systematic consolidation of these two theoretical systems, it establishes an innovative framework with prominent clinical applicability, demonstrating three distinctive advantages: Dynamic Process Management: Expands static behaviors into an ongoing cycle of monitoring, evaluation, and adjustment by adding dietary adherence and decision-making dimensions. Holistic Capacity Development: Shifts focus from resource access to self-efficacy by integrating healthcare utilization, patient role adaptation, and emotional management. Targeted Solutions: Explicitly includes patient-provider collaboration as a core dimension, addressing chronic care needs while preserving theoretical rigor. These advances enhance the models relevance by explaining dietary behaviors while addressing psychosocial challenges in chronic disease self-management for clinical applicability.

As a final conclusion, a new definition has been proposed: Nutritional self-management in chronic diseases refers to patients’ ongoing, proactive engagement in acquiring and applying evidence-based dietary knowledge under healthcare professionals’ guidance. Through this collaborative process, individuals make informed dietary adjustments, adapt to their illness role, and regulate psychological responses, thereby enhancing treatment adherence, optimizing health resources, and improving clinical outcomes.

Chronic disease nutritional self-management aligns with broader self-management principles but necessitates specific adaptations for long-term dietary challenges. Velde et al. (126) defined chronic disease self-management as a lifelong undertaking wherein patients proactively manage their conditions through informed health decisions and strategic utilization of external resources. This study posits that nutritional self-management in chronic diseases shares key attributes with general self-management concepts: (1) collaboration with healthcare professionals or trusted individuals, (2) evidence-based health decision-making, and (3) proactive engagement with one’s health status. However, distinctions emerge in: (1) Dietary adherence (specific to nutritional protocols), (2) resource acquisition, evaluation, and utilization (nutrition-focused knowledge/support systems) and (3) patient role cognition and adaptation (psychological adjustment to dietary restrictions/lifestyle changes).

Dietary management and nutritional self-management in chronic diseases are intrinsically interconnected and mutually reinforcing, synergistically improving health outcomes. Dietary management provides the behavioral foundation for nutritional self-management, whereas nutritional self-management optimizes dietary practices through personalized assessment and adaptation. Dietary adherence among chronic disease patients remains suboptimal, influenced by multifactorial determinants spanning: individual-level factors (e.g., emotional status, dietary habits, health literacy) (56, 85, 89, 127), socioenvironmental factors (e.g., family support, socioeconomic status, community resources) (79, 99, 101), and treatment-related factors (e.g., regimen complexity, adverse effects, therapeutic efficacy) (92, 93). Consequently, improving dietary adherence has emerged as a critical intervention target for optimizing nutritional self-management in chronic diseases, requiring multi-level collaborative strategies to achieve precision-based implementation.

Accumulating evidence-based medicine studies have conclusively demonstrated that effective dietary management for chronic disease patients requires the establishment of a scientific, systematic and sustainable intervention framework. The primary task involves enhancing patients’ cognitive understanding of dietary-disease relationships through structured health education, employing standardized curriculum modules integrated with motivational interviewing techniques to foster autonomous behavioral modification (73). Personalized dietary plans should be developed through shared decision-making between clinicians and patients, with ongoing adjustments guided by regular metabolic parameter monitoring and periodic clinical assessments (95). To ensure intervention sustainability, it is imperative to construct a multi-tiered support network centered on community health services, forming a collaborative supervision system incorporating professional medical teams, family members and community resources (69).

Building upon this standardized management system, digital health innovation offers a precise, sustainable solution to improve dietary adherence in chronic diseases by leveraging technology-driven, data-informed approaches. Mobile health tools powered by AI, particularly adaptive generative algorithms, deliver real-time personalized dietary plans with interactive feedback. These solutions overcome conventional drawbacks like inefficiency and limited interactivity. Emerging evidence demonstrates the broad clinical applicability of digital health interventions across diverse chronic disease populations, with multi-center studies documenting their effectiveness in achieving quantifiable improvements in treatment adherence and symptom management metrics (128–130). However, scalability challenges persist, especially in low- and middle- income countries due to low smartphone penetration (<40%) and limited health-data literacy (131–133). This technological-clinical imbalance underscores the importance of further exploring innovative “human-machine collaboration” models. Consequently, future research should prioritize elucidating differential effects of digital interventions across chronic disease subpopulations, optimizing their application in resource-limited settings, and systematically evaluating their long-term synergistic effects with core standardized management protocols (59, 63).

According to the definition, which emphasizes the ability to access, evaluate, and utilize resources, it highlights that patients should leverage external support to manage their conditions while critically assessing available information. This capability directly determines the quality of scientific decision-making in long-term disease management for patients with chronic conditions. Health information acquisition behavior refers to the behavior of people searching for, evaluating, and selecting health information, and using the relevant information to make health decisions (134). Norman defined e-health literacy as the ability to search for, analyze, and evaluate health information from electronic resources and apply the obtained information to solve their own health problems (135). The concept of nutritional self-management prioritizes three hierarchically organized competencies in patients: (1) active acquisition of evidence-based resources, (2) critical appraisal using clinical reasoning frameworks, and (3) context-driven application of knowledge through iterative learning. This framework integrates principles of clinical decision-making with self-directed learning, requiring patients to dynamically evaluate information quality and adaptively implement interventions aligned with individual health objectives.

Due to the complexity and uneven distribution of information resources, patients with chronic conditions often struggle to meet their long-term needs in areas such as medication management, nutritional guidance, and psychological support. Many lack access to reliable professional information or adequate social support, leading them to turn to non-expert sources like social media, which may result in higher-risk healthcare decisions (59, 68, 70, 87, 89). To address the aforementioned challenges, the following multidimensional strategies can be implemented: (1) policy-driven integration of evidence-based protocols into primary care with expanded insurance coverage (68, 99); (2) community-based literacy programs using culturally tailored education (79, 99); and (3) structural reforms like interdisciplinary clinics and competency training for equitable resource allocation (58, 60).

Current research on chronic diseases primarily focuses on the biomedical and behavioral factors of nutritional self-management, while paying less attention to the psychosocial mechanisms involving patients’ role cognition and adaptation (49, 51). When facing conflicts with social expectations, patients with chronic illnesses often experience cognitive role distortion, primarily manifested as role ambiguity, inter-role conflict, and role overload. These cognitive imbalances can trigger emotional stress responses such as anxiety and diminished self-efficacy, consequently impairing patients’ emotion regulation capacity and health-related decision-making processes (65, 70, 73, 78, 92, 93).

Therefore, a tripartite “medical-social-individual” collaborative intervention framework should be established to optimize role cognition and adaptation in chronic diseases through the following pathways: (1) Medical dimension: Optimize healthcare resource allocation through tiered care systems and digital solutions (62, 63). (2) Social Dimension: Utilize intelligent health communication platforms (e.g., AI-driven educational tools) to enhance public health literacy, address informational disparities, and align societal role expectations with evidence-based health practices. (3) Individual dimension: Implement capability-opportunity-motivation behavior (COM-B) based personalized nutrition programs to establish a cognition-adaptation-reinforcement cycle (51, 136).

This study systematically analyzes the “behavioral-psychological- social” multidimensional interaction mechanisms and, for the first time, conceptualizes nutrition self- management as a dynamic process comprising six core attributes, while theoretically modeling the synergistic relationships among them, thereby addressing the traditional definitions’ oversight of patients’ psychological adaptation and social role transitions. Moreover, our framework, based on the COM-B behavior diagnosis model, uncovers the nested interaction mechanism of “individual capability—external opportunity—intrinsic motivation,” particularly highlighting the pathway by which digital literacy mediates and moderates the utilization efficiency of AI-based health tools through motivation. This provides a novel evidence-based foundation for precision interventions.

## 5 Conclusion

This study adopted Walker and Avant’s concept analysis approach to systematically deconstruct the conceptual core of nutritional self-management in chronic diseases, delineating its framework: attribute identification (six core attributes including dietary adherence, patient-provider collaboration, resource utilization, decision-making, role adaptation, and emotional regulation), antecedent analysis (exploring driving factors such as sociodemographic characteristics, capability, opportunity, and motivation), consequence elaboration (individual-interpersonal/family-community/societal), and measurement indicators (adherence, nutrition literacy, and self-efficacy scales). Additionally, a tripartite case validation (typical, borderline, and contrary) is conducted to preliminarily establish an operational definition framework. Theoretically, this framework provides a conceptual foundation for standardized assessment and intervention in chronic disease nutritional management. Practically, the proposed operational definitions and measurement index system can serve as references for implementing and evaluating precision nutrition interventions. However, clinical application of this model requires further validation. Future research should focus on establishing expert consensus through Delphi methodology and conducting structural validity testing and intervention effectiveness evaluation across diverse chronic disease populations to enhance both theoretical and practical systems of nutritional self-management.

## 6 Limitations and future directions

This study employed conceptual analysis to elucidate the key attributes and influencing factors of nutritional self-management among patients with chronic diseases. The findings demonstrate that while core attributes exhibit transcultural validity, their antecedent factors vary significantly across regions. Therefore, practical implementations should incorporate localized parameter calibration. However, certain limitations regarding generalizability remain. First, the selected literature and empirical data predominantly derive from patient populations within developed healthcare systems where nutritional management resources, including access to professional dietary consultations and digital health tools, far exceed those typically available in low- to middle-income countries. Moreover, the exclusion of non-English studies may further limit cultural perspectives. Consequently, applying these findings to resource-limited settings warrants careful consideration. Notably, nutritional self-management practices exhibit significant contextual dependency, and language restrictions may obscure important cultural differences. Second, the study’s definition of self-management mainly applies to patients with basic cognitive abilities. Variations in implementation among individuals with severe complications such as cognitive impairment due to advanced kidney disease or those with low health literacy were not thoroughly examined. Additionally, most studies used cross-sectional data, limiting insights into how disease progression affects long-term nutritional management behaviors. Future research should employ multicenter collaboration to expand patient samples encompassing diverse socioeconomic backgrounds, systematically integrating multilingual data to enhance cultural representation. Concurrently, multiperspective study designs should be implemented to not only validate model generalizability but also investigate mechanistic causal pathways among attributes. Subsequent investigations should focus specifically on distinct nutritional and metabolic demands associated with major chronic diseases including diabetes mellitus, hypertension, and chronic kidney disease, conducting refined comparative analyzes of disease subtypes.
