# Supplementary material for: Nutritional self-management in chronic diseases: a conceptual analysis
Source: Front Public Health. 2025 Nov 26;13:1680903. doi: 10.3389/fpubh.2025.1680903 (PMC12689992; doi:10.3389/fpubh.2025.1680903)
Supplement: Supplementary file 1 [file Table_1.DOCX]

Supplementary Table S1

**Search strategies for all database**

| **Database** | **Search strategies** | |
| --- | --- | --- |
| **Pubmed**  ***(n=347)*** | #1 | “Self-Management”[Mesh] OR “Self Care”[Mesh] OR “Self Management” OR “Management, Self” |
|  | #2 | “Chronic Disease”[Mesh] OR “Chronic Diseases” OR “Disease, Chronic” OR “Chronic Illness” OR “Chronic Illnesses” OR “Illness, Chronic” OR “Chronically Ill” OR “Chronic Condition” OR “Chronic Conditions” OR “Condition, Chronic” |
|  | #3 | “nutrition” |
|  | #4 | “nutritional self-management” |
|  | #5 | (#1 AND #2 AND #3) OR #4 |
| **Web of Science**  ***(n=4237)*** | #1 | TS=(Self-Management OR Self Care OR Self Management OR Management, Self) |
|  | #2 | TS=(Chronic Disease OR Chronic Diseases OR Disease, Chronic OR Chronic Illness OR Chronic Illnesses OR Illness, Chronic OR Chronically Ill OR Chronic Condition OR Chronic Conditions OR Condition, Chronic) |
|  | #3 | TS=(nutrition) |
|  | #4 | TS=(nutritional self-management) |
|  | #5 | #1 AND #2 AND #3 |
|  | #6 | #4 OR #5 |
| **CINAHL**  ***(n=255)*** | #1 | Self Management OR Self Care OR Management, Self OR Self-Management Programs OR Program, Self-Management OR Self-Management Program OR Self Management Programs |
|  | #2 | Chronic Disease OR Chronic Diseases OR Disease, Chronic OR Chronic Illness OR Chronic Illnesses OR Illness, Chronic OR Chronically Ill OR Chronic Condition OR Chronic Conditions OR Condition, Chronic |
|  | #3 | nutrition |
|  | #4 | #1 AND #2 AND #3 |
|  | #5 | Nutritional self management |
|  | #6 | #4 OR #5 |
| **Embase**  ***(n=1095)*** | #1 | ('self care'/exp OR 'self care') AND [embase]/lim |
|  | #2 | ('self-management':ti,ab,kw OR 'self-nurturance':ti,ab,kw OR 'self care':ti,ab,kw OR 'self management':ti,ab,kw OR 'self treatment':ti,ab,kw OR 'self care':ti,ab,kw) AND [embase]/lim |
|  | #3 | 'chronic disease'/exp AND [embase]/lim |
|  | #4 | ('chronic illness':ti,ab,kw OR 'chronic disease':ti,ab,kw) AND [embase]/lim |
|  | #5 | ('nutrition'/exp OR 'nutrition') AND [embase]/lim |
|  | #6 | ('diet, food,':ti,ab,kw AND nutrition:ti,ab,kw OR 'nutrition council':ti,ab,kw OR 'nutrition phenomena':ti,ab,kw OR 'nutrition physiology':ti,ab,kw OR 'nutrition processes':ti,ab,kw OR 'nutrition research':ti,ab,kw OR 'nutrition research center':ti,ab,kw OR 'nutrition study':ti,ab,kw OR 'nutrition survey':ti,ab,kw OR 'nutrition surveys':ti,ab,kw OR 'nutritional physiological phenomena':ti,ab,kw OR 'nutritional physiology':ti,ab,kw OR 'nutritive solution':ti,ab,kw OR 'sports nutritional physiological phenomena':ti,ab,kw OR 'nutrition':ti,ab,kw) AND [embase]/lim |
|  | #7 | #1 OR #2 |
|  | #8 | #3 OR #4 |
|  | #9 | #5 OR #6 |
|  | #10 | #7 AND #8 AND #9 |
|  | #11 | 'nutritional self-management':ti,ab,kw AND [embase]/lim |
|  | #12 | #10 OR #11 |
|  | #13 | (#10 OR #11) AND [<1966-2025]/py |
